# Genome Sequence of *Rhizobium sullae* HCNT1 Isolated from *Hedysarum coronarium* Nodules and Featuring Peculiar Denitrification Phenotypes

**DOI:** 10.1128/genomeA.01518-17

**Published:** 2018-01-25

**Authors:** B. de Diego-Diaz, L. Treu, S. Campanaro, V. da Silva Duarte, M. Basaglia, L. Favaro, S. Casella, A. Squartini

**Affiliations:** a Department of Environmental Engineering, Technical University of Denmark, Kongens Lyngby, Denmark; b Department of Chemistry, University of Navarra, Pamplona, Navarra, Spain; c Department of Biology, University of Padua, Padua, Italy; d Department of Microbiology, Federal University of Vicosa, Vicosa, Brazil; e Department of Agronomy, Food, Natural Resources, Animals, and Environment (DAFNAE) University of Padua, Legnaro, Padua, Italy

## Abstract

The genome sequence of *Rhizobium sullae* strain HCNT1, isolated from root nodules of the legume *Hedysarum coronarium* growing in wild stands in Tuscany, Italy, is described here. Unlike other *R. sullae* strains, this isolate features a truncated denitrification pathway lacking NO/N_2_O reductase activity and displaying high sensitivity to nitrite under anaerobic conditions.

## GENOME ANNOUNCEMENT

*Rhizobium sullae* (1) is a nitrogen-fixing symbiont of the legume *Hedysarum coronarium* L. (= *Sulla coronaria*, Medik), known as French honeysuckle, or sulla. The plant is still occurring both as a cropped legume and as a spontaneous weed in Mediterranean habitats, and it is particularly tolerant to drought and alkalinity. The genome sequences of two other strains of the same bacterial species have been published, including that of the type strain IS123 (2) isolated in southern Spain from its host growing in the wild, and that of strain WSM1592 (3), isolated from cropped sulla on the island of Sardinia (Italy). The present isolate, HCNT1 (=IMAP 801, =ATCC 43676) (4), was isolated from root nodules of its host collected under spontaneous conditions in the highly calcareous pliocenic clays near Volterra (Tuscany, Italy). The strain has demonstrated peculiarities in comparison to its conspecific relatives, as it is endowed with a unique denitrifying phenotype. While capable of reducing nitrite, it appears to be incapable of coupling such a reaction to energy metabolism as true denitrifiers would (5, 6). Cell growth is also inhibited under anaerobic conditions in the presence of nitrite due to nitric oxide accumulation (7). The nitrite reductase of *R. sullae* HCNT1 has also shown to be active in reducing selenite besides nitrite (8).

Whole-genome sequencing was carried out using Illumina MiSeq sequencing technology (Ramaciotti Centre for Genomics, Sydney, Australia). The Nextera XT kit (Illumina, Inc., San Diego, CA, USA) was employed for the generation of genomic libraries. The number of paired-end reads (2 × 250 bp) obtained was 3,626,956, and this accounted for 124-fold coverage of the studied genome. Up to 99.93% of these reads were assembled into 54 scaffolds, and an *N*_50_ as high as 373,743 bp was obtained. *R. sullae* HCNT1 has been shown to have a size of 7,298,178 bp, in which the G+C content is 59.9%. Version 10.1.1 of CLC Genomics Workbench software (Qiagen Bioinformatics, Germany) was used to perform all the above-mentioned analyses.

The Rapid Annotations using Subsystems Technology (RAST) software (9) allowed the identification of 7,441 coding sequences and 50 RNAs. Thirteen prophage and phage sequences were found as well. It is also worth mentioning that virulence, disease, and defense sequences accounted for up to 99 genes in this genome, of which the vast majority (80 genes) are related to resistance to antibiotics and toxic compounds. Regarding the species characteristics, it is important to note that nitrogen metabolism is represented by 70 genes, including a copper-containing nitrite reductase (EC 1.7.2.1), 32 genes of which are related to denitrification. The genome sequence was used as input for the PHASTER software (10). This yielded two incomplete prophage regions.

A BLAST search of the 16S rRNA gene sequence against the NCBI nr database was carried out, and interestingly, the highest similarity (99% identity) was obtained with *Rhizobium* sp. strain WSM749 (not with *R. sullae*). This uncharacterized organism was isolated in Morocco from *Hedysarum flexuosum* (11), which is the closest relative of *H. coronarium* in terms of bacterial symbiont intercompatibility. In fact, isolates from *H. flexuosum* can nodulate *H. coronarium* but are ineffective in fixing nitrogen (12).

### Accession number(s).

This whole-genome shotgun project has been deposited at DDBJ/ENA/GenBank under the accession number PIQN00000000. The version described in this paper is version PIQN01000000.

## References

[1] Squartini A, Struffi P, Döring H, Selenska-Pobell S, Tola E, Giacomini A, Vendramin E, Velázquez E, Mateos PF, Martínez-Molina E, Dazzo FB, Casella S, Nuti MP 2002 *Rhizobium sullae* sp. nov. (formerly ‘*Rhizobium hedysari*’), the root-nodule microsymbiont of *Hedysarum coronarium* L. Int J Syst Evol Microbiol 52:1267–1276. doi:10.1099/00207713-52-4-1267. 12148639

[2] Sablok G, Rosselli R, Seeman T, van Velzen R, Polone E, Giacomini A, La Porta N, Geurts R, Muresu R, Squartini A 2017 Draft genome sequence of the nitrogen-fixing *Rhizobium sullae* type strain IS123^T^ focusing on the key genes for symbiosis with its host *Hedysarum coronarium* L. Front Microbiol 8:1348. doi:10.3389/fmicb.2017.01348. 28798728 PMC5526965

[3] Yates R, Howieson J, De Meyer SE, Tian R, Seshadri R, Pati A, Woyke T, Markowitz V, Ivanova N, Kyrpides N, Loi A, Nutt B, Garau G, Sulas L, Reeve W 2015 High-quality permanent draft genome sequence of *Rhizobium sullae* strain WSM1592; a *Hedysarum coronarium* microsymbiont from Sassari, Italy. Stand Genomic Sci 10:44. doi:10.1186/s40793-015-0020-2. 26380632 PMC4572446

[4] Casella S, Gault RR, Reynolds KC, Dyson JR, Brockwell J 1984 Nodulation studies on legumes exotic to Australia: *Hedysarum coronarium*. FEMS Microbiol Lett 22:37–45. doi:10.1111/j.1574-6968.1984.tb00350.x.

[5] Casella S, Shapleigh JP, Payne WJ 1986 Nitrite reduction in *Rhizobium* “*hedysari*” strain HCNT1. Arch Microbiol 146:233–238. doi:10.1007/BF00403222.

[6] Casella S, Shapleigh JP, Payne WJ 1988 Nitrite reduction in bacteroids of *Rhizobium* “*hedysari*” strain HCNT1. Arch Microbiol 149:384–388.

[7] Toffanin A, Wu Q, Maskus M, Caselia S, Abruña HD, Shapleigh JP 1996 Characterization of the gene encoding nitrite reductase and the physiological consequences of its expression in the nondenitrifying *Rhizobium* “*hedysari*” strain HCNT1. Appl Environ Microbiol 62:4019–4025. 8899992 10.1128/aem.62.11.4019-4025.1996 PMC168221

[8] Basaglia M, Toffanin A, Baldan E, Bottegal M, Shapleigh JP, Casella S 2007 Selenite-reducing capacity of the copper-containing nitrite reductase of *Rhizobium sullae*. FEMS Microbiol Lett 269:124–130. doi:10.1111/j.1574-6968.2006.00617.x. 17227457

[9] Aziz RK, Bartels D, Best AA, DeJongh M, Disz T, Edwards RA, Formsma K, Gerdes S, Glass EM, Kubal M, Meyer F, Olsen GJ, Olson R, Osterman AL, Overbeek RA, McNeil LK, Paarmann D, Paczian T, Parrello B, Pusch GD, Reich C, Stevens R, Vassieva O, Vonstein V, Wilke A, Zagnitko O 2008 The RAST server: rapid annotations using subsystems technology. BMC Genomics 9:75. doi:10.1186/1471-2164-9-75. 18261238 PMC2265698

[10] Arndt D, Grant JR, Marcu A, Sajed T, Pon A, Liang Y, Wishart DS 2016 PHASTER: a better, faster version of the PHAST phage search tool. Nucleic Acids Res 44:W16–W21. doi:10.1093/nar/gkw387. 27141966 PMC4987931

[11] Kishinevsky BD, Nandasena KG, Yates RJ, Nemas C, Howieson JG 2003 Phenotypic and genetic diversity among rhizobia isolated from three *Hedysarum* species: *H*. *spinosissimum*, *H*. *coronarium* and *H*. *flexuosum*. Plant Soil 251:143–153. doi:10.1023/A:1022967213088.

[12] Glatzle A, Schulte-Batenbrock T, Brockwell J 1986 Symbiotic incompatibility between two forage species of *Hedysarum* grown in Morocco, and their homologous rhizobia. FEMS Microbiol Lett 37:39–43. doi:10.1111/j.1574-6968.1986.tb01763.x.

